# Interface Gain-of-Function Mutations in TLR7 Cause Systemic and Neuro-inflammatory Disease

**DOI:** 10.1007/s10875-024-01660-6

**Published:** 2024-02-07

**Authors:** Clémence David, Mihaly Badonyi, Robin Kechiche, Antonella Insalaco, Marco Zecca, Fabrizio De Benedetti, Simona Orcesi, Luisa Chiapparini, Patrizia Comoli, Silvia Federici, Marco Gattorno, Monia Ginevrino, Elisa Giorgio, Valentina Matteo, Patricia Moran-Alvarez, Davide Politano, Giusi Prencipe, Fabio Sirchia, Stefano Volpi, Cécile Masson, Gillian I. Rice, Marie-Louise Frémond, Alice Lepelley, Joseph A. Marsh, Yanick J. Crow

**Affiliations:** 1 Laboratory of Neurogenetics and NeuroinflammationImagine Institute, INSERM UMR1163, Paris, France; 2 MRC Human Genetics Unit, Institute of Genetics and Cancer, University of Edinburgh, Edinburgh, UK; 3 Department of Paediatric Hematology-Immunology and Rheumatology, Necker-Enfants Malades Hospital, AP-HP, Paris, France; 4 Division of Rheumatology, IRCCS Ospedale Pediatrico Bambino Gesù, Rome, Italy; 5 Pediatric Haematology/Oncology, Fondazione IRCCS Policlinico San Matteo, Pavia, Italy; 6 Department of Brain and Behavioural Sciences, University of Pavia, Pavia, Italy; 7 Child Neurology and Psychiatry Unit, IRCCS Mondino Foundation, Pavia, Italy; 8 Neuroradiology Unit, Fondazione IRCCS Policlinico San Matteo, Pavia, Italy; 9 Cell Factory, Fondazione IRCCS Policlinico San Matteo, Pavia, Italy; 10 UOC Reumatologia E Malattie Autoinfiammatorie, IRCCS Istituto Giannina Gaslini, Genoa, Italy; 11 Translational Cytogenomics Research Unit, Bambino Gesù Children’s Hospital, IRCCS, Rome, Italy; 12 Department of Molecular Medicine, University of Pavia, Pavia, Italy; 13 Medical Genetics Unit, IRCCS Mondino Foundation, Pavia, Italy; 14 Laboratory of Immuno-Rheumatology, Bambino Gesù Children’s Hospital, IRCCS, Rome, Italy; 15 Dipartimento Di Neuroscienze, Riabilitazione, Oftalmologia, Genetica e Scienze Materno-Infantili (DINOGMI), Università Degli Studi Di Genova, Genoa, Italy; 16 Bioinformatics Core Facility, Paris-Cité University-Structure Fédérative de Recherche Necker, INSERM US24/CNRS UMS3633, Paris, France; 17 Division of Evolution and Genomic Sciences, School of Biological Sciences, Faculty of Biology, Medicine and Health, University of Manchester, Manchester Academic Health Science Centre, Manchester, UK; 18 Reference Center for Rheumatic, AutoImmune and Systemic Diseases in Children (RAISE), Paris, France; 19 University Paris Cité, Paris, France

**Keywords:** TLR7, systemic lupus erythematosus, stem cell transplantation

## Abstract

TLR7 recognizes pathogen-derived single-stranded RNA (ssRNA), a function integral to the innate immune response to viral infection. Notably, TLR7 can also recognize self-derived ssRNA, with gain-of-function mutations in human TLR7 recently identified to cause both early-onset systemic lupus erythematosus (SLE) and neuromyelitis optica. Here, we describe two novel mutations in TLR7, F507S and L528I. While the L528I substitution arose de novo, the F507S mutation was present in three individuals from the same family, including a severely affected male, notably given that the TLR7 gene is situated on the X chromosome and that all other cases so far described have been female. The observation of mutations at residues 507 and 528 of TLR7 indicates the importance of the TLR7 dimerization interface in maintaining immune homeostasis, where we predict that altered homo-dimerization enhances TLR7 signaling. Finally, while mutations in TLR7 can result in SLE-like disease, our data suggest a broader phenotypic spectrum associated with TLR7 gain-of-function, including significant neurological involvement.

**Supplementary Information:**

The online version contains supplementary material available at 10.1007/s10875-024-01660-6.

## Introduction

Systemic lupus erythematosus (SLE) describes a heterogeneous set of clinical phenotypes associated with type I interferon (IFN) signaling upregulation and the presence of autoantibodies targeting nuclear autoantigens [1]. Familial aggregation and higher concordance rates between monozygotic versus dizygotic twins suggest a major hereditary component, with rare monogenic forms of SLE providing important insights into disease pathogenesis [2, 3]. Expressed in sentinel cells such as macrophages and dendritic cells, Toll-like receptors (TLRs) are a family of single-pass membrane-spanning proteins that engage structurally conserved microbial features as part of a coordinated innate and adaptive immune response to pathogens. Endosomal TLR3, TLR7/8, and TLR9 traffic from the ER to the endosomal compartment where they sense nucleic acids, respectively, dsRNA, ssRNA, and CpG DNA. Inherited TLR3 deficiency underlies herpes simplex encephalitis [4] and severe influenza or COVID-19 pneumonia [5], and inherited TLR7 deficiency predisposes to critical COVID-19 pneumonia [6]. Importantly, TLR7/8 and TLR9 can signal upon sensing both viral- and self-derived nucleic acid [7]. Reflective of the latter situation, disease in murine models of SLE is attenuated in animals deficient for TLR7 [8], while SLE-like pathology is driven by TLR7 overexpression [9]. Further, gain-of-function (GOF) mutations in human TLR7 were recently identified to cause severe autoimmune phenotypes, specifically, SLE and neuromyelitis optica [10]. These observations indicate the importance of endosomal TLR signaling in immunological homeostasis. Here, we describe two novel mutations in TLR7, causing a spectrum of systemic and neuro-inflammatory diseases.

## Materials and Methods

### Samples Obtained from Patients

Samples were obtained from the probands and parents with written informed consent. The study was approved by the Comité de Protection des Personnes (ID-RCB/EUDRACT: 2014-A01017-40, revalidated in 2022).

### Genetic Studies

DNA was extracted from whole blood using standard methods. In family AGS571, a Twist Custom Panel Kit (Twist Bioscience), comprising around 8000 disease-causing genes, was used for library preparation, and a NovaSeq6000 platform (Illumina) for sequencing. In family AGS3740, exome sequencing was performed on genomic DNA using a SureSelect Human All Exon Kit (Agilent Technologies) for targeted enrichment and Illumina HiSeq2000 for sequencing. Variants were assessed using the in silico programs SIFT (http://sift.jcvi.org) and Polyphen2 (http://genetics.bwh.harvard.edu/pph2/). Population allele frequencies were obtained from the gnomAD database (http://gnomad.broadinstitute.org). Sanger sequencing was performed to confirm the TLR7 variant identified in family AGS3740. The reference sequence used for primer design and nucleotide numbering was TLR7: ENST00000380659.4/NM_016562.4; NP_057646.1.

### Protein Structure Modeling

We searched the Protein Data Bank [11] on 16/11/2023 for protein structures that have > 90% homology with TLR7 (UniProt ID: Q9NYK1) over a stretch of at least 50 amino acids. Twenty-three structures were analyzed with FoldX 5.0 [12] to estimate the change in Gibbs free energy (ΔΔG) of the missense variants as the average of 3 replicates. The FoldX “RepairPDB” command was run before modeling the mutations. The buried surface area was defined as the solvent-accessible surface area between the monomer and the complex and was calculated at the residue level using AREAIMOL from the CCP4 software suite [13]. Structure visualization was performed with PyMOL [14].

### Cell Culture

Human embryonic kidney (HEK) 293 T cells were grown in DMEM (GIBCO) supplemented with 10% (v/v) fetal bovine serum (GIBCO) and maintained at 37 °C with 5% CO_2_.

### Plasmids

pCMV6 vector encoding wild-type (WT) TLR7 was used as the parental vector for mutagenesis [6]. Mutant plasmids of TLR7 were generated via site-directed mutagenesis using the Q5 kit (E0554S; New England Biolabs) according to the manufacturer’s instructions and using oligonucleotides listed in the table below. NEB 5-alpha competent *Escherichia coli* were transformed with the ligated product, and colonies were screened by sequencing for the presence of the desired variants. pcDNA 3.1™ vector encoding V5-tagged human WT UNC93B1 has been described previously [5].

## Primers Used for Site-Directed Mutagenesis of TLR7

**Table:** 

Variation(s)	Forward primer	Reverse primer
F507S	TAG TAT ATT TTC TGT CAA GTC CTC	TTT TTA CTT AGA TCC AAG GTC
F507L	AGT ATA TTT TTG GTC AAG TCC TC	ATT TTT ACT TAG ATC CAA GGT C
L528I	TGT CAG GAA ATA TCA TTA GCC AAA C	GAT TCA GGC ATT TGA GGA AAG

### Luciferase Reporter Assay

For each condition, 2.10^4^ HEK293T cells per well were plated in a 96-well plate in duplicate and co-transfected with a plasmid containing the *Firefly* luciferase gene under the control of the human NF-κB promoter (pGL4.32, Promega), a plasmid constitutively expressing the *Renilla* luciferase gene for normalization (pRL-SV40, Promega), as well as a plasmid encoding WT, empty vector (EV) or variant TLR7 and a plasmid encoding UNC93B1, using the TransIT®-293 Transfection Reagent (Mirus, # MIR2700) according to the manufacturer’s instructions. After incubation for 24 h, cells were left either unstimulated or stimulated with the TLR7 agonist R848 0.01, 0.1, and 1 µg/mL for 24 h. Cells were then lysed, and luciferase levels were measured with the Dual-Luciferase® Reporter assay system (Promega, #E1980) according to the manufacturer’s protocol. Luminescence intensity was acquired on an Infinite® F200 Pro microplate reader (TECAN). Firefly luciferase activity values were normalized against Renilla luciferase activity before further data processing (see figure legend).

## Results

### Clinical, Laboratory, and Radiological Data

#### Family AGS571

**Fig. 1:**
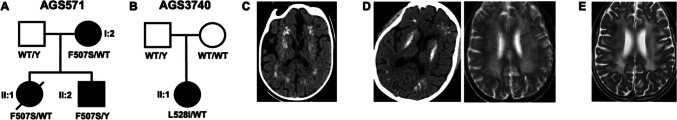
Genetic and clinical data. Family pedigrees (**A** AGS571; **B** AGS3740) where an affected individual harbors a rare non-synonymous missense substitution in TLR7, in either the hemizygous (male) or heterozygous (female) state. Circles and squares indicate female and male family members, respectively. Filled shapes indicate affected status. The line across II:1 in AGS571 indicates deceased status. WT = wild-type. **C** Cranial CT in AGS571 II:2 at the age of < 1 year. **D** Cranial CT (left) and axial T2-weighted MRI (right) of AGS3740 II:1 at age 10 years. **E** Progression of high signal in the deep white matter of AGS3740 II:1 by age 11 years (1 year after hematopoietic stem cell transplantation)

The proband (II:1) was a female born to non-consanguineous parents of Italian ancestry (Fig. 1A). She was well until age 4 years, with normal physical and neurological development (Table 1, Table S1). At that age, she presented with fever, malar rash, and cytopenia (lymphopenia and anemia). One year later, she began to experience severe headaches and hallucinations. She was subsequently diagnosed with extensive cerebral vasculitis and suffered a cerebrovascular infarct, considered secondary to high positivity for antiphospholipid antibodies, resulting in a major neurological deficit. Widespread micro-calcifications were noted at that time on cranial computed tomography. Titers of antinuclear antibodies (ANA) and anti-dsDNA were elevated, and there was C3 and C4 hypocomplementemia. She subsequently developed a class III/IV lupus nephritis and intestinal ischemia. She was treated with steroids, hydroxychloroquine, mycophenolate mofetil, and fondaparinux (the latter as secondary thromboprophylaxis). During follow-up, she continued to show low levels of complement, but autoantibody screening was negative. She died of an acute myocardial infarction at the age of 17 years.

**Table 1:** Patients harboring heterozygous TLR7 missense substitutions identified in this study

	Nucleotide	Amino acid	Inheritance	gnomAD v4	Phenotype
AGS571	c.1520 T > C	F507S	Maternal in children	0/112,190^a^	Proband: SLE (fever, malar rash, cytopenia, raised AABs, low complement, LN), cerebral vasculitis with CVA, cerebral calcification, MI (causing death aged 17 years) Sibling: refractory epilepsy, cerebral calcification, fever, rash, leukopenia, low complement (but AAB negative), dystonia, severe DD Mother: malar rash, arterial thrombosis of leg in adulthood, raised AABs, low complement
AGS3740	c.1582C > A	L528I	De novo	0/640,595^b^	Mild motor developmental delay, immune thrombocytopaenia and anemia (leading to HSCT), stereotypic movement disorder, cerebral WMD with atrophy and calcification progressing post-transplant

*AAB* autoantibody, *CVA* cerebrovascular accident, *DD* developmental delay, *HSCT* hematopoietic stem cell transplantation, *LN* lupus nephritis, *MI* myocardial infarction, *SLE* systemic lupus erythematosus, *WMD* white matter disease

^a^The denominator (112,190) is derived from a p.Phe507LeufsTer16 recorded on gnomAD. Further, note that a p.Ile505Thr and a p.Lys509Glu are each seen once on gnomAD out of 457,192 and 456,952 alleles respectively

^b^The denominator (640,595) is derived from a p.Leu528Leu recorded on gnomAD

After a term pregnancy and normal delivery (birth weight 2.63 kg, head circumference 49 cm), the proband’s brother (II:2) presented with refractory epilepsy starting on the second day of life. Neurological work-up demonstrated the presence of basal ganglia calcification and bilateral reduction of supratentorial white matter on cranial imaging (Fig. 1C). The child developed recurrent episodes of fever and rash on the limbs, trunk, and face from the age of 3 months. Laboratory tests revealed leukopenia and low C3 and C4. C-reactive protein and erythrocyte sedimentation rate were not elevated, and autoantibody screening (ANA, anti-dsDNA, IgG/IgM anticardiolipin, IgG/IgM anti-B2-glycoprotein and lupus anticoagulant) was negative. Screening for congenital infection and metabolic disorders was non-contributory. Immunosuppression with glucocorticoids, hydroxychloroquine, mycophenolate mofetil, and the JAK1/2 inhibitor ruxolitinib was started with good response (resolution of fever, hematological and skin manifestations), but ruxolitinib was subsequently withdrawn due to recurrent viral infection. Currently, the boy is 3 years of age and cannot sit, walk or talk. Visual evoked potentials showed a bilateral increase in cortical latency with a normal auditory brainstem response. The patient has no dysphagia or respiratory problems. He exhibits a dystonic movement disorder. His current neurological state appears to be stable.

The mother of II:1 and II:2 had a history of malar rash and ANA positivity identified at age 12 years. At 24 years of age, she was diagnosed with immune thrombocytopenic purpura and treated with steroids for 2 years. Aged 44 years she experienced a major arterial thrombotic event in the right lower limb, at which time proteinuria was noted. Immunological work-up showed low levels of complement in the absence of ANA, ENA, and antiphospholipid autoantibodies.

Interferon signaling in whole blood tested in the proband at age 7 years was elevated, with an interferon score of 14.416 (normal < 2.446) (Fig. S1A). Further testing in all three affected individuals was also grossly elevated (Fig. S1B).

#### Family AGS3740

This now 11-year-old female (II:1) was born to non-consanguineous parents of Italian ancestry with no family history of note (Fig. 1B). She was delivered vaginally at 36 weeks gestation weighing 3.3 kg. During infancy, she exhibited mild motor developmental delay, achieving complete head control at 5 months, independent sitting at 8 months, and independent walking at 18 months. At 1 year of age, she developed immune thrombocytopenia refractory to multiple therapies (steroids, intravenous immunoglobulins, mycophenolate mofetil, rituximab, eltrombopag) except sirolimus and, later, autoimmune hemolytic anemia (negative Coombs reaction, positive anti-SSA), leading to a diagnosis of Evans-like/SLE-like syndrome.

At age 8 years, she was referred to pediatric neurology because of a stereotypic movement disorder (i.e., head nodding), present since age 18 months. Cranial MRI revealed multiple hyperintense foci in cortico-subcortical areas, mainly parasagittal in the frontal and parietal lobes, also involving the temporal lobes and periventricular white matter (Fig. 1D). Serial brain MRIs from 8 years of age, including after transplantation (Fig. 1E), demonstrated a progressive, diffuse leukoencephalopathy of the deep white matter, associated with brain atrophy and both supra- and infra-tentorial scattered calcifications (stone-like in the basal ganglia), in the absence of a clear worsening of neurological status.

By the age of 10 years, both hematological conditions had become refractory to all previous therapies (including the addition of bortezomib), and life-threatening, with platelet counts consistently < 10 × 10^9^/L and hemoglobin < 7 g/dl, prompting peripheral blood hematopoietic stem cell transplantation (HSCT) from an HLA-matched unrelated donor. The conditioning regimen included thiotepa, treosulfan, fludarabine, and rabbit anti-T lymphocyte globulin and graft-versus-host disease (GVHD) prophylaxis comprising cyclosporine A and mycophenolate mofetil. HSCT was successful, with prompt achievement of 100% donor chimerism in peripheral blood mononuclear cells as early as day + 10 after HSCT. Neutrophil engraftment (first of 3 consecutive days with neutrophils > 0.5 × 10^9^/L) was achieved on day + 11, while platelet engraftment (first of 3 consecutive days with platelets > 50 × 10^9^/L) was achieved on day + 105 post-HSCT. Grade I acute cutaneous GVHD developed on day + 29 but was completely resolved with topical steroids. Six months after transplantation, the patient developed skin maculopathy and panniculitis, both thought to be due to limited chronic GVHD. Histological confirmation was obtained 10 months after HSCT. Initial treatment included imatinib and rituximab, subsequently changed to ruxolitinib, started 16 months after HSCT. At the last hematological examination, 22 months after HSCT, the patient had 100% donor chimerism and a completely normal blood count. After 6 months of treatment with ruxolitinib, an initial improvement in chronic cutaneous GVHD was observed.

On the last neurological examination, at the age of 11 years and 10 months, there was a slight reduction in muscle strength and processing speed and some hypokinesia.

### Molecular Data

**Fig. 2:**
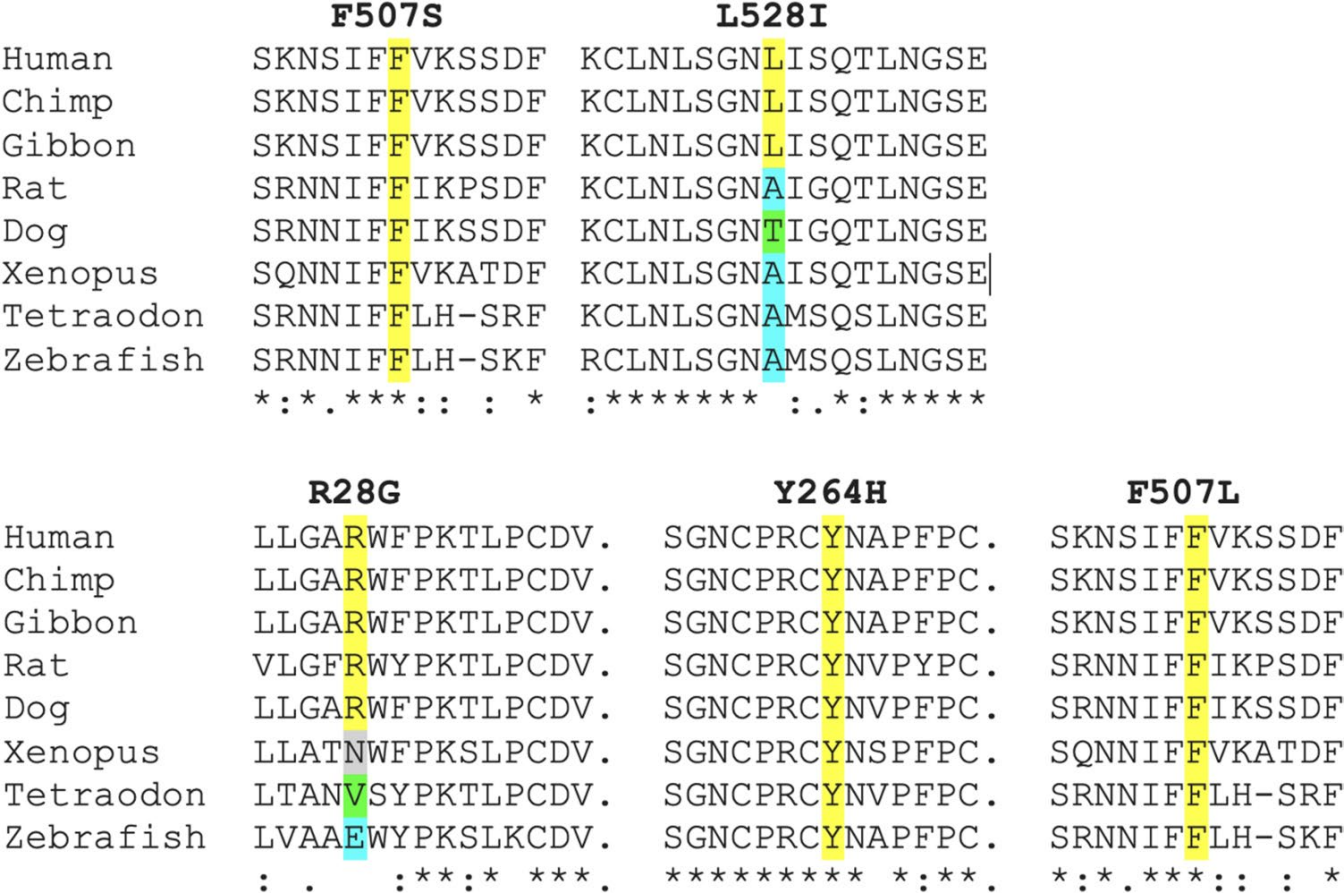
Clustal Omega alignment of TLR7. Clustal Omega alignment of TLR7 with identified non-synonymous missense substitutions highlighted. Top: substitutions identified in this study. Bottom: substitutions identified in Brown et al. Alignments are based on the human transcript of TLR7: ENST00000380659.4/NM_016562.4; NP_057646.1

In AGS571, both affected children were identified to carry a maternally inherited p.(Phe507Ser) (F507S) (c.1520 T > C) substitution in TLR7 (Fig. S2A). The phenylalanine at residue 507 is highly conserved (Fig. 2), and the F507S substitution is not present on gnomAD v4 (0 of 112,190 alleles). The same residue was reported to be mutated to a leucine (F507L) in Brown et al. [10] (again, maternally inherited).

In AGS3740, the affected child was shown to harbor a de novo p.(Leu528Ile) (L528I) (c.1582C > A) substitution in TLR7 (Fig. S2B). The leucine at residue 528 is not particularly well conserved (Fig. 2). However, this variant is not present on gnomAD v4 (0 of 640,595 alleles).

### In Vitro Data

**Fig. 3:**
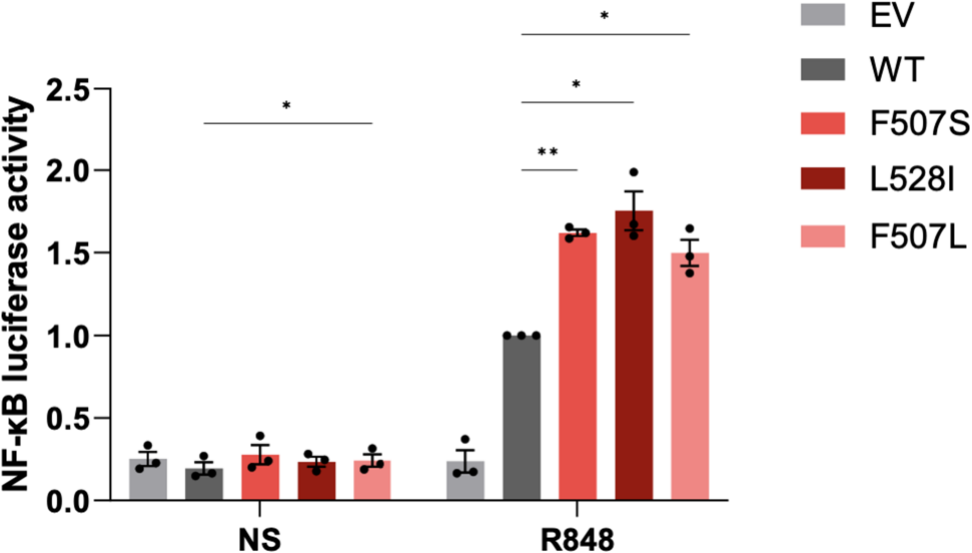
In vitro assessment of TLR7 signaling. NF-κB luciferase reporter activity following co-transfection of HEK293T cells with empty vector (EV), wild-type (WT) TLR7 or variants of TLR7, and UNC93B1 plasmid and stimulation with R848 0.1 µg/mL. Data are normalized to WT response to R848. Mean ± SEM of *n* = 3 experiments. Two-way ANOVA with Dunnett’s post hoc test: **p < 0.01, *p < 0.5. NS: non-stimulated

To investigate the effect of these two missense substitutions on TLR signaling, we expressed WT and variant (c.1520 T > C (F507S) and c.1582C > A (L528I)) *TLR7* in HEK293T cells, which lack TLRs, using an NF-κB luciferase reporter as the read-out. We measured reporter activity following stimulation with the TLR7 agonist R848. Both the F507S and the L528I variants were found to confer a gain-of-function in TLR7 activity (Fig. 3, Fig. S3), similar to two recently described gain-of-function mutations (F507L and Y264H) in TLR7 [10].

### Protein Structure Modeling

**Fig. 4:**
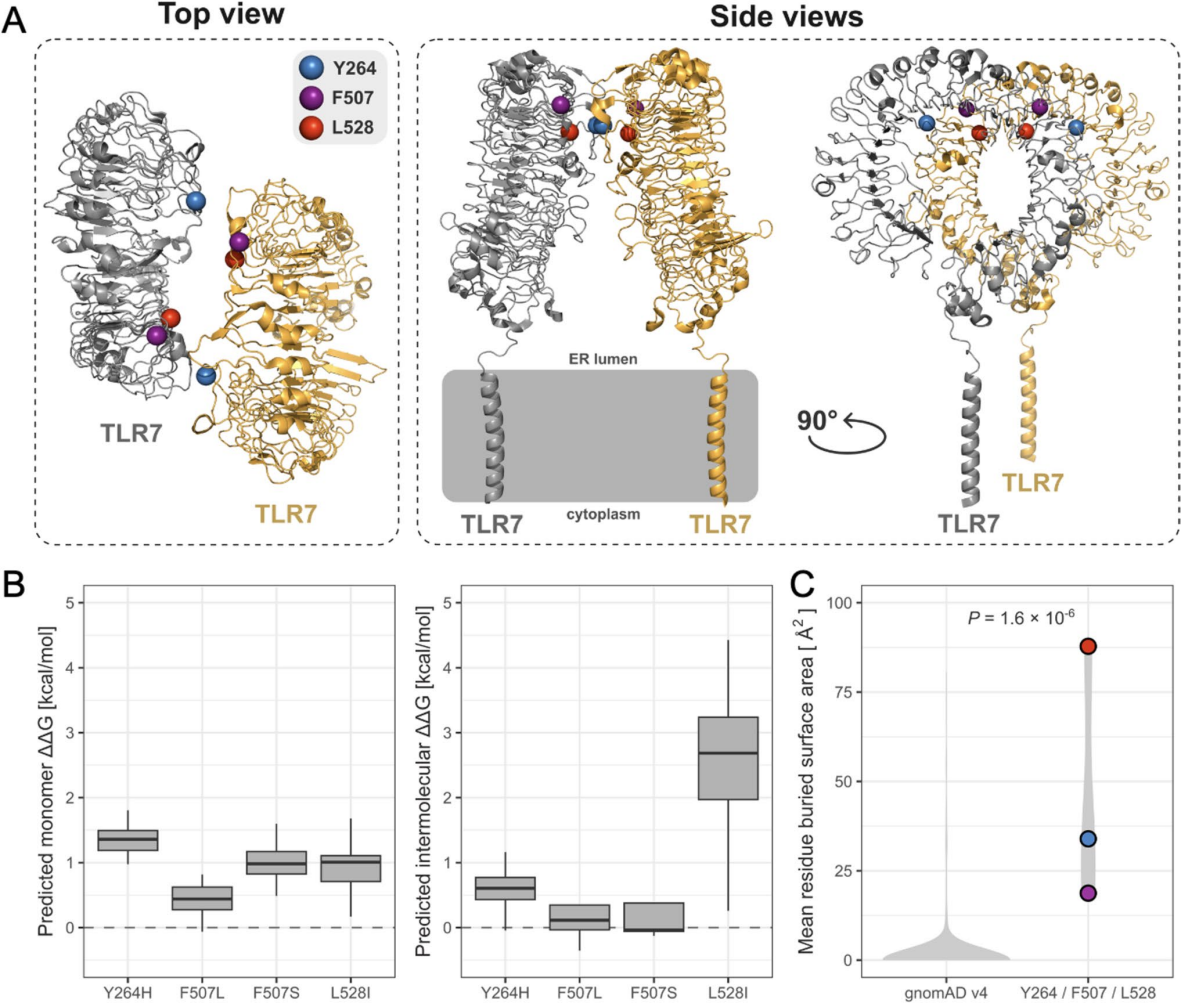
Modeling of TLR7 and assessment of identified variants. **A** Top view and side views of TLR7 structure based upon PDB ID 7cyn, with the location of indicated amino acids (variants at F507 and L528 found in this study) highlighted. **B** Predicted change in the Gibbs free energy of folding (ΔΔG) of TLR7 variants measured in monomeric TLR7 structures (left). Predicted intermolecular ΔΔG defined as the difference between the ΔΔG of the TLR7 monomer and the dimer (right). Box, 25th and 75th percentiles; middle line, median; whiskers, 1.5 times the interquartile range. **C** Violin plot showing the average buried surface areas of residues at which gnomAD (v4) variants are recorded in TLR7 versus the 3 residues of the 4 variants identified in this study or by Brown et al. [10]. *P*-value was calculated with a two-sided Wilcoxon rank-sum test (right)

The F507, L528, and Y264 residues all lie at the endosomal leucine-rich repeat (LRR) domain of TLR7 (Fig. 4A). We used a meta-structural analysis approach to explore the likely structural impacts of missense variants by modeling their energetic effects (ΔΔG) across 23 different experimentally determined TLR7 structures, considering both the stability of the monomeric protein and their intermolecular effects on the TLR7 homodimer (Fig. 4B). All variants were predicted to have minimal effects on protein stability, based on previous observations suggesting ~ 1.5 kcal/mol as an approximate ΔΔG threshold above which mutations are less likely to be tolerated in monomers [15, 16]. In contrast, when considering intermolecular effects, L528I is predicted to be highly disruptive to the TLR7 dimerization interface, with much smaller intermolecular effects predicted for the other variants. However, despite the small predicted intermolecular effects of these variants, it is notable that the F507, L528, and Y264 residues are situated directly at the homodimeric interface, compared to < 10% (35/365) of missense variants recorded in gnomAD v4 (*P* = 0.0001, Fisher’s exact test). Moreover, the residues associated with pathogenic variants tend to bury significantly more intermolecular surface area in the dimer interface than the sites of the gnomAD variants (Fig. 4C). Given that dimerization of TLR7 is necessary for its activation [17], this suggests that intermolecular effects are crucial for pathogenicity.

## Discussion

TLRs are type I transmembrane proteins with an extracellular LRR domain, a single transmembrane helix, and a cytoplasmic Toll/interleukin-1 receptor (TIR) domain [18]. On recognition of ligand by the LRR domain, inactive monomeric or preformed dimeric TLRs are induced to form a face-to-face activated dimer [19]. In this way, TIR domains are brought into proximity with each other, thereby recruiting downstream adaptor proteins to initiate transcriptionally-driven immune responses. TLR7 contains two distinct ligand-binding sites, recognizing ssRNAs in the form of degradation products, nucleosides, and oligoribonucleotides [20]. Site 1, which is highly conserved between TLR7 and TLR8, recognizes both nucleosides (guanosine for TLR7, uridine for TLR8) and nucleoside analogs and is essential for receptor dimerization. In contrast, site 2 is not conserved, is spatially distinct between TLR7 and TLR8, and appears to play an auxiliary role in receptor dimerization by enhancing the binding affinities of site 1 ligands.

Here, we describe two novel mutations in TLR7, F507S and L528I. While the L528I mutation is predicted to impact intermolecular contacts at the TLR7 dimer interface, this does not necessarily mean that dimerization will be completely disrupted. Notably, there is considerable variability in the precise orientation of the TLR7 dimer in the available experimental structures, suggesting that it might be robust to a single amino acid change. Comparable to the F507L mutation described by Brown et al. [10], the F507S substitution is predicted to have a minimal effect on monomeric and dimeric TLR7 structures. Neither of the L528 and F507 residues are in direct contact with the guanosine (or other RNA ligand) binding site in any structure. Brown et al. described a Y264H substitution to map to ligand-binding site 1 [10], suggesting an enhanced affinity of the Y264H for guanosine. However, since Y264 lies at the TLR7 dimer interface, it remains possible that this substitution might also play a role in homo-dimerization. We highlight this point by noting that four (F507L, Y264H, F507S, L528I) of the five mutations in human TLR7 described to date, not including R28G (possibly a signal peptide-related variant: Brown et al. [10]), occur directly at the homodimer interface in multiple structures. Of possible further interest, although TLR7-TLR7 molecules are in contact with one another, no noticeable interactions (hydrogen bond, intimate contact, etc.), that might be important in dimer stabilization, were observed in the structure reported by Ishida et al. [17], indicating that dimerization is loosely achieved (and perhaps partly explaining why the isolated endosomal domain of TLR7 mainly exists as a monomer in solution [21]). All told, these observations suggest the importance of the TLR7 interface in maintaining immune homeostasis, where we predict that altered homo-dimerization enhances TLR7 signaling. It will be interesting to map the position of further mutations in human TLR7 as they are reported.

While GOF mutations in TLR7 can result in a phenotype consistent with SLE, our data emphasize a broader spectrum of disease, including significant neurological involvement. Thus, the child with a de novo Y264H mutation described by Brown et al. [10] demonstrated recurrent hemichorea requiring treatment with haloperidol (Table S2), and in a second case reported by the same authors, a child with a maternally inherited F507L mutation, the phenotype was exclusively neurological, consistent with neuromyelitis optica and positive for AQP4 autoantibodies (a phenomenon recognized in the context of enhanced type I interferon signaling [22]). In our series of four affected patients, we observed cerebral vasculitis and cerebral calcification in the female proband in family AGS571 and developmental delay, epilepsy, and marked intracranial calcification in her brother. Further, white matter disease, atrophy, and calcification were seen in the proband in family AGS3740, which was progressive despite an apparently excellent response of life-threatening immune cytopenias to HSCT.

Finally, given that the TLR7 gene is situated on the X chromosome, the observation of a severely affected male in our series is notable, with all other cases so far described being female (and where variable levels of X-inactivation might affect the clinical phenotype).

### Supplementary Information

Below is the link to the electronic supplementary material.

Supplementary file1 (DOCX 494 KB)

## Data Availability

No datasets were generated or analysed during the current study.
