# Supplementary material for: Interface Gain-of-Function Mutations in TLR7 Cause Systemic and Neuro-inflammatory Disease
Source: J Clin Immunol. 2024 Feb 7;44(2):60. doi: 10.1007/s10875-024-01660-6 (PMC10850255; doi:10.1007/s10875-024-01660-6)
Supplement: Supplementary file 1 — Supplementary file1 (DOCX 494 KB) [file 10875_2024_1660_MOESM1_ESM.docx]

**Supplementary file**

**Table S1.** **Clinical and laboratory features of cases reported in this study**.

|  | **AGS571 II:1** | **AGS571 II:2** | **AGS571 I:1** | **AGS3740 II:1** |
| --- | --- | --- | --- | --- |
| **Age at onset** | 4 years | 2 days | 12 years | 1 year |
| **Gender** | Female | Male | Female | Female |
| **Hematologic** | Anaemia, lymphopenia | Leukopenia | Thrombocytopenia, anaemia | Thrombocytopenia, anaemia |
| **Fever** | Yes | Yes | No | No |
| **Cutaneous** | Malar rash | Malar rash, rash on limbs and trunk | Malar rash | Panniculitis |
| **Neurologic** | Cerebral vasculitis, cerebral infarct, cerebral calcification | Refractory epilepsy, cerebral calcification, dystonia, severe developmental delay | No | Mild motor developmental delay, stereotypic movement disorder, cerebral calcification, cerebral atrophy and progressive leukoencephalopathy |
| **Renal** | Class III/IV lupus nephritis | No | NA | No |
| **Other** | MI aged 17 years |  |  | Maculopathy and panniculitis post-transplant |
| **Immunological parameters** | Low complement; AAB screen positive (ANA+, anti-dsDNA+, aCL+, aB2GPI+, LA+) | Low complement, AAB screen negative | Low complement, ANA + (subsequent AAB screen negative off treatment) | Negative Coombs reaction, positive anti-ANA, negative anti-dsDNA |
| **Interferon signature** | Elevated | Elevated | Elevated | Not assessed pre-transplant; negative post-transplant |
| **Current status / age at death** | Died of MI aged 17 years | Alive age 45 years | Alive age 3 years | Alive age 11 years |

AAB: autoantibody; aB2GP: anti-B2-glycoprotein antibodies; aCL: anticardiolipin antibodies; anti-dsDNA: anti-double-stranded DNA antibodies; ANA: antinuclear antibodies; LA: lupus anticoagulant; MI: myocardial infarction

**Table S2. Patients harbouring TLR7 missense substitutions described by Brown et al. (Nature 2022;605:349-356)**

|  | **Nucleotide** | **Amino acid** | **Inheritance** | **gnomAD v4** | **Phenotype** |
| --- | --- | --- | --- | --- | --- |
| Family A | c.790T>C | p.Tyr264His (Y264H) | De novo | 0 | SLE plus relapsing hemichorea |
| Family B | c.1521T>G | p.Phe507Leu (F507L) | Maternal | 0 | **Proband**: Relapsing ON +TM +AQP4 AAB (NMO); **Mother**: ‘hemiplegic CP’, and SLE in 20s |
| Family C | c.82A>G | p.Arg28Gly (R28G) | Not defined | 0 | SLE |

AAB: autoantibody; CP: cerebral palsy; NMO: neuromyelitis optica; ON: optic neuritis; SLE: systemic lupus erythematosus; TM: transverse myelitis

**Clinical data of patients described by Brown et al. (Nature 2022;605:349-356)**

**Brown (A.II.1); Female. De novo Y264H**: Presented at age 7 years with refractory immune thrombocytopenia and hemichorea. Elevated ANAs and hypocomplementemia. She also suffered from inflammatory arthralgias and constitutional symptoms. Renal involvement a year later when she presented with a hypertensive crisis (140-150 / 80 mmHg > P99 for her height and sex). Has suffered intermittent episodes of chorea, treated with haloperidol.

**Brown (B.II.1); Female. Maternally inherited F507L**: Presented at age 9 years with relapsing optic neuritis and transverse myelitis. Found to have NMO/AQP4-IgG in serum and CSF. ANA profile demonstrated positive ANA (1:320, speckled) without other autoantibodies. First treated with rituximab but had additional exacerbations of NMO leading to treatment with IVIG and mycophenolate mofetil. Mother has hemiplegic cerebral palsy of unclear aetiology and developed SLE in her mid-20s.

**Brown (C.I.1); Female. R28G (inheritance not determined)**: Presented at age 18 years with malar rash, joint pain, Raynaud's phenomenon, alopecia, fever and oral ulcers. Positive ANA (1:1280), U1RNP, SSA-Ro52, SSA-Ro60, and dsDNA (Farr 42.55U/ml). Full blood count showed lymphocytopenia (0.26 * 10^9/ml) and thrombocytopenia (55 * 10^9/ml). She also had low C3 [0.785g/L (0.9-1.8)]. CNS evaluation could not be performed. Active episode treated with methylprednisolone 80mg IV.

**Figure S1. Expression of interferon stimulated genes (ISGs) measured in AGS571 II:1 at the age of 7 years (A), and in all three affected individuals from this same family (B).**

**A.**

**
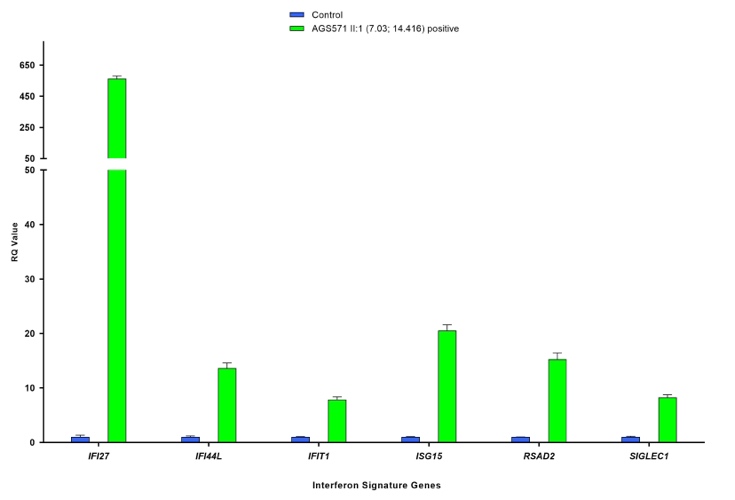
**The expression in whole blood of six representative ISGs (green bars, patient data; blue bars, composite data for 29 control individuals). The first number in brackets is the decimalised age at sampling, the second number is the interferon score (Normal < 2.466) (methodology described in Rice et al. 2013;12:1159-69).

**B**.

**
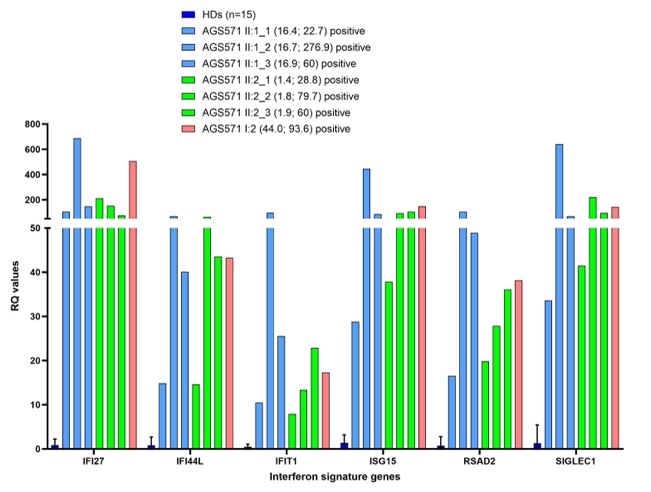
**

The expression in whole blood of six representative ISGs in all three affected family members in AGS571. The first number in brackets is the decimalised age at sampling, the second number is the interferon score (normal < 2.05 - based on data from 15 healthy controls).

**Figure S2. Sequencing data relating to AGS571 (A) and AGS3740 (B), demonstrating the c.1520T>C and c.1582C>A nucleotide substitutions respectively.**

**A**.


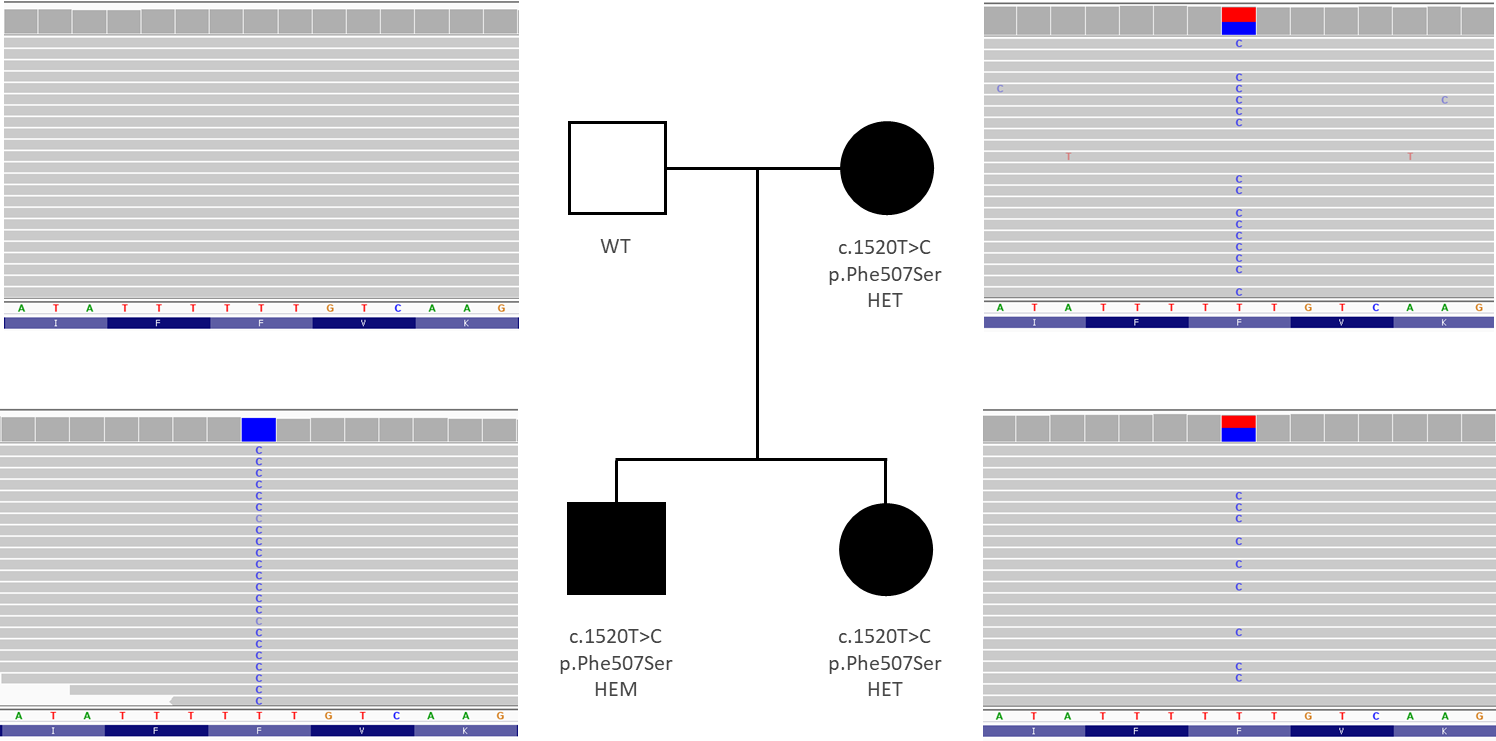


**B**.


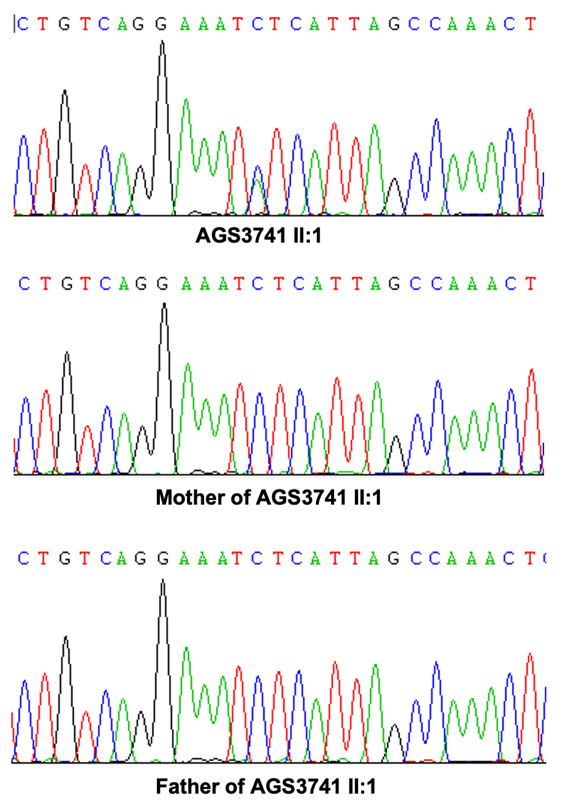


**Figure S3. In vitro data using different doses (0.01, 0.1 and 1 µg/mL) of R848 in the context of the two mutations reported in our paper (F507S, L528I), and the F507L mutation described by Brown et al. (Nature. 2022;605:349-56).**


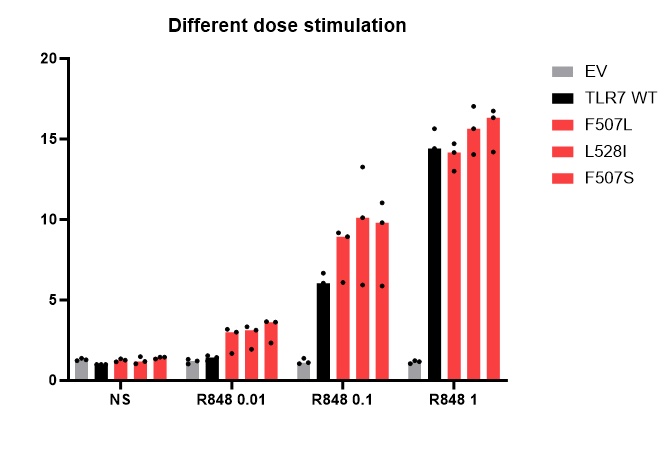


NS, non-stimulated; EV, empty vector; WT, wild-type.
